# Feasibility of a lifestyle intervention in early pregnancy to prevent deterioration of glucose tolerance

**DOI:** 10.1186/1471-2458-11-179

**Published:** 2011-03-24

**Authors:** Eeva AL Korpi-Hyövälti, David E Laaksonen, Ursula S Schwab, Tarja H Vanhapiha, Kristiina R Vihla, Seppo T Heinonen, Leo K Niskanen

**Affiliations:** 1 Department of Internal Medicine, Seinäjoki Central Hospital, Seinäjoki, Finland; 2 Department of Medicine, Kuopio University Hospital, Kuopio, Finland; 3 Institute of Biomedicine, Physiology, University of Eastern Finland, Kuopio Campus, Finland; 4 Department of Clinical Nutrition, School of Public Health and Clinical Nutrition, University of Eastern Finland, Kuopio Campus, Finland; 5 Kauhajoki Health Center Social Welfares, Kauhajoki, Finland; 6 Lapua Health Center, Lapua, Finland; 7 Department of Obstetrics and Gynecology, Kuopio University Hospital, Finland

**Keywords:** gestational diabetes mellitus, lifestyle intervention, oral glucose tolerance test, insulin treatment

## Abstract

**Background:**

In conjunction with the growing prevalence of obesity and the older age of pregnant women gestational diabetes (GDM) is a major health problem.

The aim of the study was to evaluate if a lifestyle intervention since early pregnancy is feasible in improving the glucose tolerance of women at a high-risk for GDM in Finland.

**Methods:**

A 75-g oral glucose tolerance test (OGTT) was performed in early pregnancy (n = 102). Women at high risk for GDM (n = 54) were randomized at weeks 8-12 from Apr 2005 to May 2006 to a lifestyle intervention group (n = 27) or to a close follow-up group (n = 27). An OGTT was performed again at weeks 26-28 for the lifestyle intervention and close follow-up groups.

**Results:**

The values of the OGTT during the second trimester did not differ between the lifestyle intervention and close follow-up groups. In the lifestyle intervention group three women had GDM in the second trimester and respectively one woman in the close follow up group. Insulin therapy was not required in both groups. The intervention resulted in somewhat lower weight gain 11.4 ± 6.0 kg vs. 13.9 ± 5.1 kg, p = 0.062, adjusted by the prepregnancy weight.

**Conclusions:**

Early intervention with an OGTT and simple lifestyle advice is feasible. A more intensive lifestyle intervention did not offer additional benefits with respect to glucose tolerance, although it tended to ameliorate the weight gain.

**Trial Registration:**

ClinicalTrials.gov: NCT01130012

## Background

Management for women with gestational diabetes mellitus (GDM) consists of dietary counselling and physical exercise, and for those women who fail to maintain glycemic goals, insulin therapy [1]. Type 2 diabetes can be prevented or delayed by lifestyle changes, including increased physical activity, improvements in diet and modest weight loss in high-risk individuals [2,3]. There is little evidence demonstrating that GDM can be prevented by lifestyle changes in women at a high risk for GDM. Dempsey et al. found women most active within the first 20 weeks of pregnancy were half as likely to develop GDM [4].

GDM is defined as carbohydrate intolerance of varying severity with onset or first recognition during pregnancy [5,6]. Type 2 diabetes and GDM have similarities: both are characterized by a strong family history, overweight, insulin resistance, and lack of compensatory pancreatic insulin secretion in demanding hormonal circumstances [7,8]. GDM is usually diagnosed between 24-28 weeks of pregnancy [9].

The occurrence of GDM varies from 2.2 to 8.8% depending on the diagnostic criteria used and population studied [10]. Like type 2 diabetes, the prevalence of GDM is increasing at an alarming rate worldwide [11]. There has been a long-standing controversy regarding whom and how to screen for GDM. Like many other European countries, Finland focuses screening on high-risk groups in the beginning of the third trimester 26-28 weeks [12-14].

We therefore carried out a randomized controlled trial in 54 pregnant women at high risk for GDM. We compared the effectiveness of early intensive lifestyle intervention to a single session lifestyle advice combined with a close follow-up. The hypothesis of this feasibility study was that early screening with an oral glucose tolerance test (OGTT) and lifestyle treatment since early pregnancy can improve glucose tolerance and decrease the incidence of GDM and related perinatal complications.

## Methods

The study was an open multicenter randomized and controlled study with two rural municipalities: Kauhajoki and Lapua. The recruitment started in April 2005 and ended in May 2006. Deliveries occurred from Nov 2005 to Dec 2006. The protocol was approved by the ethics committee of South Ostrobothnia Hospital District in Seinäjoki, Finland. It was in accordance with Helsinki Declaration. All women participating in the trial gave written informed consent.

The health care nurses gave women counselling about healthy lifestyle in the beginning of pregnancy. The dietary and exercise advice were provided both verbally and in writing. Women were advised to stop alcohol intake and smoking.

### Randomisation

**Figure 1:**
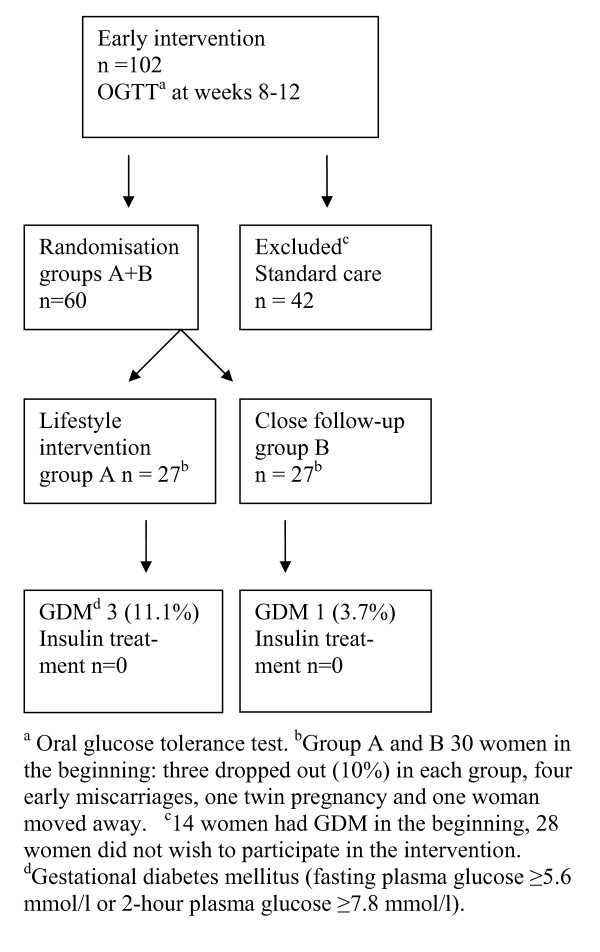
**Formation of the study populations**.

A 2-hour OGTT was offered to all women in the first contact with maternal health care units during gestational weeks 8-12. If the women had one or more risk factors (BMI > 25 kg/m^2^, previous history of GDM or birth of child > 4.5 kg, age > 40 years, family history of diabetes i.e. parents, children, siblings or grandparents) or the venous plasma glucose concentration after 12 hours fasting in the morning was 4.8-5.5mmol/l and 2-hour OGTT plasma glucose < 7.8mmol/l, they were recruited to the intervention (Figure 1).

**Figure 2:**
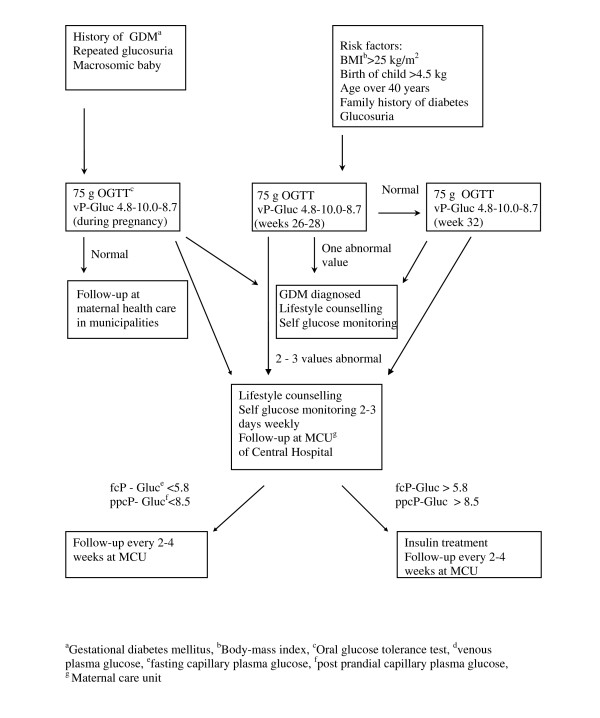
**Screening and follow-up of the gestational diabetes in Finland 2005**.

These high-risk women were randomly assigned to the lifestyle intervention group (n = 27) or to the close follow-up group (n = 27) by the study physician in the Central Hospital with the use of a computed randomisation list (Figure 1). We randomized 60 women, three dropped out (10%) in each group (four early miscarriages, one twin pregnancy and one woman moved away). The health care nurses who scheduled the study visits did not have access to the randomisation list. We excluded women (n = 14) who were diagnosed as having GDM in this study and women who had risk factors for GDM or whose fasting venous plasma glucose was 4.8-5.5mmol/l but who for personal or professional reasons did not wish to participate in the trial (n = 28). Obstetricians who were not study physicians made decisions concerning the beginning of insulin treatment, if the glucose targets were not achieved (fasting capillary glucose > 5.8 mmol/l or postprandial capillary glucose > 8.5 mmol/l during self-monitoring, Figure 2).

### Diet counselling in the lifestyle intervention group

Based on the general dietary recommendations of the Diabetes and Nutrition Study Group (DNSG) of European Association for the Study of Diabetes [15] for diabetes and the Finnish Diabetes Prevention Study [2], the goals of the diet in this study were carbohydrate 50-55 energy percent (E%), fibre 15 g/1000 kcal, fat 30 E%, saturated fat < 10 E% and protein 15-20 E%. Women were encouraged to eat a diet rich in vegetables, berries and fruits, and to use low-fat dairy products, low-fat meat, soft margarines and vegetable oils and whole-grain products. Recommendation for energy intake was 30 kcal/kg/day for normal weight women and 25 kcal/kg/day for overweight women.

The nurse in the health care centres had on average 13 appointments with the intervention women. The intervention women had no instruction on self glucose monitoring before GDM was diagnosed. The nutritionist gave dietary advice tailored to each subject individually six times. The three-factor eating questionnaire was used in the beginning of pregnancy and at weeks 36-40 [16].

### Exercise counselling in the lifestyle intervention group

We encouraged moderate-intensity physical exercise during pregnancy. The formula (220-age) × 0.65-0.75 was used to estimate the heart rate goals for moderate-intensity exercise [17]. Other measures of exercise intensity included the "talk test" [18], i.e. exercise at an intensity in which the woman is able to maintain a conversation during exercise. A target rating of 12 to 14 on Borg's scale of perceived exertion [19] was also used.

The pregnant women had six appointments with the physiotherapist. During sessions the physiotherapist motivated the women individually to continue exercising during pregnancy or to start exercising, and gave also written instructions for exercise and self care. When starting an aerobic exercise program, previously sedentary women were instructed to begin with 15 minutes of continuous exercise three times a week [20], increasing gradually to 30 minute sessions four times a week [21]. The goal of the exercise intervention was 30 minutes of daily physical activity, if the woman previously exercised < 2.5 hours per week, and 45 minutes activity [22], if the woman already engaged in 2.5 hours or more per week of physical activity. Recommendable types of exercise were brisk walking, Nordic walking, swimming, cycling and cross country skiing [23]. If the BMI of a woman was > 30 kg/m^2 ^and the woman was not physically active before pregnancy, the exercise was started with 15 minutes per day three times a week [20,22]. Women were offered both aerobics classes and aquafit classes weekly.

### Close follow-up group

The women were informed of the results of the OGTT during gestational weeks 8-12. All women were also given general information on diet and physical activity to decrease the risk of GDM during pregnancy. Dietary information was collected three times during pregnancy. They returned a self reported exercise history and a monthly questionnaire of physical activity. Otherwise, the women were followed up in the prenatal clinic of the municipal health centre at one-month intervals according to standard care in Finland (Figure 2) [24].

### Definition of GDM

The GDM criteria were modified from the World Health Organization as a fasting plasma glucose 5.6mmol/l or 2-hour plasma glucose 7.8mmol/l [6].

### Screening for GDM

A 2-hour OGTT containing 75 g glucose was offered to all women in the early intervention groups during gestational weeks 8-12 (Table 1). The OGTT was repeated during gestational weeks 26-28.

**Table 1:** Research design and methods

Counselling and follow-up	Lifestyle intervention (n = 27)^a^	Close follow-up (n = 27)^a^
Lifestyle counselling (nurse)	+	+

OGTT^b ^(weeks 8-12)	+	+

SGM^c^	-	-

Diet reporting^d^	3 times	3 times

TFEQ^e^	2 times	2 times

Counseling(clinical nutritionist)	6 times	-

Exercise history	+	+

Exercise reporting diaries monthly	6 times	6 times

Exercise counselling (physiotherapist)	6 times, twice in groups	-

OGTT (weeks 26-28)	+	+

GDM^f^-counselling (nurse) and SGM	+	+

^a^60 women randomized in the beginning: three dropped out (10%) in each group (4 early miscarriages, one twin pregnancy and one woman moved away).

^b^Oral glucose tolerance test

^c^Self glucose monitoring

^d^4-day food records at baseline, at weeks 26-28 and in the end of pregnancy

^e^The three-factor eating questionnaire [16]

^f^Gestational diabetes mellitus

### Examinations

A nurse measured height, weight, and blood pressure of women at the first appointment during gestational weeks 8-12. Blood pressure was measured twice on the right arm with the subject in a sitting position after 10 min of rest, using a standard automatic sphygmomanometer. Newborns and placentae were weighed immediately after delivery. The data of singleton pregnancies and deliveries were extracted from the medical records in maternal health care units and in the Central Hospital of Seinäjoki.

A 75 g 2-hour oral glucose tolerance test after overnight fasting for 12 hour was performed at gestational weeks 8-12 and at weeks 26-28 with measurement of plasma glucose at 0, 1 and 2 hours. Plasma glucose was determined immediately with a photometric hexokinase assay from samples drawn into a fluoride-citrate tube (Abbot Laboratories, Abbot Park IL) in the Central Hospital of Seinäjoki.

### Statistical analysis

Statistical analyses were based on the intention to treat. The final analyses were conducted using SPSS for Windows version 15.0 (SPSS Inc, Chicago, Illinois). Differences between the groups were analysed by Student's t-test for continuous variables, and the chi-square test and Fisher's exact test for categorical variables. Statistical significance was set at the 95% level (p < 0.05).

## Results

### Baseline characteristics of the study groups

There were no statistically significant differences in baseline measures between the lifestyle intervention (n = 27) and the close follow-up (n = 27) groups (Table 2).

**Table 2:** Baseline characteristics of the women (mean ± SD or numbers, percentage in parentheses).

Characteristic	Lifestyle intervention n = 27	Close follow-up n = 27	p value^a^
Age	29.1 ± 5.4	29.8 ± 5.4	NS

Primiparous	13 (50)	17 (63)	NS

Body-mass index (kg/m^2^)	27.3 ± 6.0	25.5 ± 3.4	NS

Prepregnancy weight (kg)	76.6 ± 16.1	69.6 ± 9.7	0.061

Previous Cesarean delivery	3 (12)	1 (3.7)	NS

Risk factors	25 (86.2)	21 (72.4)	NS

BMI > 25 (kg/m^2^)	18 (60)	17 (56.7)	NS

Previous birth of child > 4.5 kg	0	1 (3.4)	NS

Age > 40 years	0	1 (3.3)	NS

Previous history of GDM	5 (16.7)	1 (3.4)	NS

Family history of diabetes	13 (43.3)	12 (41.4)	NS

Prepregnancy smoking	1 (3.7)	2 (7.4)	NS

Higher educational status	5 (18.5)	12 (44.4)	0.080

Office or service work	13 (46.4)	18 (64.3)	NS

Fasting glucose (mmol/l)	5.0 ± 0.3	4.9 ± 0.3	NS

OGTT 1-hour glucose (mmol/l)	6.4 ± 1.4	6.0 ± 1.1	NS

OGTT 2-hour glucose (mmol/l)	5.6 ± 1.0	5.4 ± 1.0	NS

Area under the curve (mmol/l/2 h)	11.7 ± 1.7	11.2 ± 1.5	NS

Systolic blood pressure (mmHg)	117.3 ± 10.3	119.4 ± 8.9	NS

Diastolic blood pressure (mmHg)	72.9 ± 7.9	70.9 ± 7.4	NS

^a^p value: Student's t-test for independent samples, chi-square or Fisher's exact test.

### Glucose tolerance at weeks 26-28

There was no difference between the randomized groups in the change in glucose values from baseline to gestational weeks 26-28 during the 2-hour OGTT. There was also no difference between the randomized groups in glucose tolerance at weeks 26-28. GDM was diagnosed in three of the lifestyle intervention group and in one of the close follow-up group. None of them required insulin therapy (Table 3).

**Table 3:** Weight gain, oral glucose tolerance test at weeks 26-28 and requirement of insulin therapy (mean ± SD or numbers, percentage in parentheses).

Outcomes	Lifestyle intervention group n = 27^a^	Close follow-up group n = 27	p value^b^
Total weight gain (kg)	11.4 ± 6.0	13.9 ± 5.1	NS

Weight at the end of pregnancy ( kg)	88.6 ± 14.4	84.1 ± 11.3	NS

Fasting glucose (mmol/l)	4.6 ± 0.4	4.4 ± 0.3	NS

OGTT 1-hour glucose (mmol/l)	6.9 ± 1.7	6.9 ± 1.7	NS

OGTT 2-hour glucose (mmol/l)	6.1 ± 1.4	6.0 ± 1.2	NS

Area under the curve (mmol/l/2 h)	12.3 ± 2.4	12.1 ± 2.2	NS

Gestational diabetes	3 (11.1)	1 (3.7)	NS

Insulin therapy	0	0	NS

^a^An OGTT was carried out in 26 (96.3%) women in the lifestyle intervention group.

^b^p value: Student's t-test for independent samples or Fisher's exact test was used.

### Maternal outcomes

The intervention resulted in somewhat lower weight gain during pregnancy (11.4 ± 6.0 kg vs. 13.9 ± 5.1 kg, p = 0.062, adjusted by the prepregnancy weight).

There was no statistically significant difference between the randomised groups in terms of pre-eclampsia, induction of labor, lacerations, Cesarean deliveries (data not shown).

### Newborn outcomes

The mean birth weight was greater 3871 ± 567 g in the lifestyle intervention group (p = 0.047, adjusted by the prepregnancy weight of the women) compared with the close follow-up group 3491 ± 573 g. The mean birth weight was 3564 g in the period 1996-2000 in Finland [25]. There was no difference in macrosomia (p = 0.480, adjusted by the prepregnancy weight of the women) between the groups.

There was no statistically significant difference between the randomized groups in terms of gestational age, admissions to neonatal intensive care unit, jaundice requiring phototherapy or respiratory distress (data not shown).

## Discussion

Early intervention with an OGTT and lifestyle advice in high-risk mothers is feasible. More intensive lifestyle advice was not more effective than close follow-up. However, weight gain during pregnancy tended to be lower in the intensive group. The rate of neonatal complications in the study groups was similar.

Somewhat surprisingly, the intensive lifestyle intervention and the close follow-up were equally effective with respect to glucose tolerance and GDM in the current study. High-risk pregnant women may be particularly receptive to lifestyle advice. On the other hand a part of women are not able to change their lifestyle or the maintenance of applied eating or physical activity habits are short sighted even during pregnancy. Small group differences at randomization may also have affected the results. Prepregnancy weight in the lifestyle intervention group tended to be higher compared with the close follow-up group, and all women weighing over 100 kg were in the intervention group. The women in the close follow-up group tended to have a higher educational status (p = 0.080).

Few studies have assessed the role of intervention early in pregnancy on the prevention or treatment of GDM. In the study by Bartha et al. women with an early diagnosis and treatment of gestational diabetes represented a high-risk group with a worse prognosis regarding pregnancy complications and outcomes [26]. Callaway recruited 25 obese women at 12 weeks' gestation in a randomized controlled study. They received an individualized exercise program with an energy expenditure (EE) goal of 900 kcal/week. This intervention was feasible and prompted a modest increase in physical activity [27]. The study was underpowered to detect an effect on GDM.

We used GDM criteria modified from the WHO definition (fasting plasma glucose 5.6mmol/l or 2-hour glucose 7.8mmol/l) [6]. Interestingly, we found 14 cases of GDM already in the beginning of pregnancy, suggesting that they had prepregnancy impairment of glucose metabolism. Treatment for GDM was initiated in early pregnancy for these women, and four normalized their glucose tolerance and only three required insulin therapy later in their pregnancy. The prevalence of GDM was 12.0% (31/258) in study municipalities and at the same time period in two neighboring municipalities without early intervention 14.5% (26/181). The difference was greater concerning the treatment with insulin: 2.3% (6/258) of pregnant women in study municipalities had insulin treatment compared with 8.3% (15/181) of women with the standard care. Screening of glucose tolerance among high-risk, especially overweight women and lifestyle advice in early pregnancy may be an effective way to improve glucose tolerance in high-risk women in later pregnancy.

When an OGTT is performed very early in the pregnancy it is worth noting, that fasting glucose concentrations reach their nadir at 12 weeks of gestation and remain at this level until delivery [28].

The encouraging finding was that the intervention resulted in somewhat, albeit not statistically significantly lower weight gain as compared with the close follow-up group. However, the prepregnancy weight tended to be higher (p = 0.061) in the intervention group than in the close follow-up group. Obese women on average gain less weight during pregnancy than women with normal weight [29]. In the analyses, however, we adjusted for prepregnancy weight. In Finland, the mean weight gain during pregnancy has increased from 13.3 kg in 1960 to 14.3 kg in 2000 [30]. In the same period the prepregnancy weight has increased from 57.6 kg to 65.5 kg [30]. In the study municipalities the prepregnancy weight was 69.1 ± 15.1 kg.

In large cohorts of pregnant women, there has been a continuous relationship between mother's glycemia and newborn birth weight below standard cut-off levels for GDM [31,32]. Our study group is too small to conclude from pregnancy outcomes.

One of the strengths of this feasibility study is that it was performed in a community-based setting in a rural area, and the intervention was conducted with little or no extra resources. Although the results of the study were encouraging, there were obvious weaknesses. First, the study had a relatively small number of participants. Second, the interventions seemed to be so "effective" that the number of cases of GDM in the randomized groups of high-risk women was very small, only four out of 54 women. Third we had no international consensus of criteria for GDM in the year 2005. Finnish threshold value, especially the fasting glucose 4.8mmol/l, for the diagnosis of GDM was too low in early pregnancy. This is the reason why we used the modified WHO definition for the early intervention groups. This complicated the study setting. This study was carried out as a feasibility study. The results should be viewed against this background and need to be corroborated in larger studies.

## Conclusions

In this community-based intervention study, our findings suggest that early intervention with an OGTT and simple lifestyle advice is feasible. More intensive lifestyle intervention does not seem to have marked additional benefits. Concentration on high-risk, especially overweight women may be important and useful. Larger trials are nonetheless needed.

## Abbreviations

AUC: area under the curve; BMI: body mass index; GDM: gestational diabetes mellitus; DNSG: Diabetes and Nutrition Study Group; DPS: Diabetes Prevention Study; EASD: European Association for the Study of Diabetes; EE: energy expenditure; E%: Energy percent; fcP-Gluc: fasting capillary plasma glucose; HAPO: Hyperglycemia and Adverse Pregnancy Outcomes; MCU: Maternal Care Unit; NICU: Neonatal Intensive Care Unit; OGTT: oral glucose tolerance test; ppcP-Gluc: post prandial capillary plasma glucose; SGM: self glucose monitoring; vP-Gluc: venous plasma glucose; WHO: World Health Organization

## Competing interests

The authors declare that they have no competing interests.

## Authors' contributions

EK-H participated in the design of the study, acquisition of the data, performed the statistical analysis and drafted the manuscript. DEL, LN, US and SH had substantial contributions to conception and design of the study and they helped to draft the manuscript. TV and KV participated in data collection. All authors read and approved the final manuscript.

## Pre-publication history

The pre-publication history for this paper can be accessed here:

http://www.biomedcentral.com/1471-2458/11/179/prepub
